# AGE-RELATED DECLINES IN SOCIAL COGNITIVE PROCESSES OF OLDER ADULTS

**DOI:** 10.1093/geroni/igz038.3232

**Published:** 2019-11-08

**Authors:** W Quin Yow, Jiawen Lee, Xiaoqian Li

**Affiliations:** Singapore University of Technology & Design, Singapore, Singapore

## Abstract

Despite current literature suggesting that various social cognitive processes seem to be impaired in late adulthood, e.g., processing of social gaze cues, the trajectory decline of social cognition in late adulthood is not well understood (e.g., Grainger et al., 2018; Paal & Bereczkei, 2007). As part of a multi-institutional research project, we began to systematically investigate whether there is age-related decline in older adults’ ability to infer others’ mental states, integrate multiple referential cues, and identify emotional states of others using prosodic cues. Sixteen older adults aged 71-85, of which 9 were cognitively healthy and 7 with mild-to-moderate dementia, and 7 younger adults aged 19-37 underwent three tasks. In a theory-of-mind story task, participants answered true/false questions about the beliefs of the protagonists in the stories. A cue integration task assessed participants’ ability to integrate the experimenter’s gaze and semantic cues to identify a referent object. In an emotion-prosody task, participants judged whether the speaker sounded happy or sad in low-pass filtered audio. Non-parametric tests revealed that younger adults outperformed both groups of older adults (both ps=.001) in inferring the protagonists’ beliefs in the stories. Younger adults were also better and more accurate than both groups of older adults in integrating cues to identify the referent object and in using prosodic cues to identify emotional states respectively (ps<.001). Both groups of older adults did not differ significantly from each other in the tasks. These findings provide emerging and important insights into the decline of social cognitive processes in late adulthood.

